# Effects of gastrointestinal delivery of non-caloric tastants on energy intake: a systematic review and meta-analysis

**DOI:** 10.1007/s00394-021-02485-4

**Published:** 2021-02-08

**Authors:** Tim Klaassen, Daniel Keszthelyi, Freddy J. Troost, Aalt Bast, Adrian A. M. Masclee

**Affiliations:** 1 Division of Gastroenterology-Hepatology, Department of Internal Medicine, School of Nutrition and Translational Research in Metabolism (NUTRIM), Maastricht University Medical Center+, P.O. Box 5800, 6202 AZ Maastricht, The Netherlands; 2 Food Innovation and Health, Center for Healthy Eating and Food Innovation, Maastricht University, 5911 AA Venlo, The Netherlands

**Keywords:** Taste, Energy intake, Eating behavior, Gastrointestinal, Satiety, Motility

## Abstract

**Purpose:**

Taste receptors are expressed throughout the gastrointestinal tract. The activation of post-oral taste receptors using tastants could provide a non-invasive treatment option in combating the obesity epidemic. The aim of this review was to examine the effect of post-oral delivery of non-caloric tastants on eating behavior reflected by primary outcome energy intake and secondary outcomes GI symptoms and perceptions and potential underlying mechanisms. This review was conducted according to the PRISMA guidelines for systematic reviews.

**Methods:**

A systematic literature search of the Cochrane, PubMed, Embase, and Medline databases was performed. This systematic review and meta-analysis was registered in the PROSPERO database on 26 February 2020 (ID: CRD42020171182). Two researchers independently screened 11,912 articles and extracted information from 19 articles. If at least two studies investigated the effect of the same taste compound on primary outcome energy intake, a meta-analysis was performed to determine pooled effect sizes.

**Results:**

Nineteen papers including healthy volunteers were included. In the 19 papers analyzed, effects of various tastants were investigated in healthy volunteers. Most extensively investigated were bitter tastants. The meta-analysis of effects of bitter tastants showed a significant reduction in energy intake of 54.62 kcal (95% CI − 78.54 to − 30.69, *p* = 0.0014).

**Conclusions:**

Bitter stimuli are most potent to influence eating behavior. Energy intake decreased after post-oral delivery of bitter tastants. This highlights the potential of a preventive role of bitter tastants in battling the obesity epidemic.

**Supplementary Information:**

The online version contains supplementary material available at 10.1007/s00394-021-02485-4.

## Introduction

There are at least five prototypical basic tastes that can be distinguished by humans: sweet, sour, bitter, salty, and umami. More recent studies have pointed to the existence of other basic tastes (i.e., fat and starch) [1, 2] as well as taste disorders such as metallic taste in cancer patients treated with chemotherapy [3]. Moreover, there is a phenomenon known as chemesthesis, which refers to chemical sensations that are perceived as warmth, heat, irritation, cooling, or pungency [4]. A prototypical pungent stimulus is capsaicin, resulting in a sizable number of studies investigating the effects of capsaicin as a weight loss intervention [5, 6].

As far as the prototypical basic tastes are concerned, these can be sensed by taste buds present on the tongue. Ion channels mediate the sensing of salty and sour taste, whereas sensing sweet, bitter and umami taste is mediated by two families of taste receptors. Taste receptor family 1 (TAS1) generally senses sweet and umami taste and taste receptor family 2 (TAS2) primarily senses bitter taste [7]. It is hypothesized that these prototypical tastes exist to predict the type of food that is ingested (i.e., sweet for saccharides, umami for glutamate, and bitter for potential toxic substances) [8]. However, it should be noted that several studies show that the negative affective response to bitter can be decoupled by, for instance, the positive response to caffeine [9, 10]. In addition, several studies have shown that the activation of oral taste receptors can result in the release of gastrointestinal (GI) peptides such as peptide YY (PYY), glucagon-like peptide 1 (GLP-1), and cholecystokinin (CCK) [11, 12]. These GI peptides have been shown to influence eating behavior by reducing appetite sensations and food intake after intravenous administration [13–18].

Taste receptors are not only present on the tongue but are expressed throughout the entire human gut [19–22]. In the GI-tract, entero-endocrine cells (EECs) are co-localized with these taste receptors. The in vitro studies have shown that activation of these taste-receptors results in the release of GI peptides [23–25].

Activation of taste receptors can be elicited using non-caloric tastants. Taste receptor activation using non-caloric tastants to influence eating behavior is potentially considered as a non-invasive treatment option in combating the obesity epidemic [26]. This concept deserves further evaluation. To date, a significant number of papers [27–45] describing the effects of post-oral delivery of non-caloric tastants (i.e., exposure to tastants anywhere distal to the oral cavity) on eating behavior, and in particular energy intake, have been published. However, due to inconsistent results reported in these papers, the effect of post-oral delivery of non-caloric tastants on eating behavior remains unclear and a detailed overview of the literature on the effects of post-oral delivery of non-caloric tastants on eating behavior is lacking. Therefore, we conducted a systematic review and meta-analysis using the PRISMA guidelines for systematic reviews. In order to keep this review and meta-analysis concise, we focused on the prototypical basic tastes; novel taste entities and chemesthesis were deemed out of scope.

Our aim was to systematically address randomized controlled trials investigating the effects of post-oral delivery of prototypical non-caloric tastants versus placebo on energy intake in healthy volunteers. Our secondary aims were to evaluate the effects of post-oral delivery of non-caloric tastants versus placebo on GI symptoms and perceptions and potential underlying mechanisms in healthy volunteers. We hypothesized that post-oral delivery of non-caloric tastants results in decreased energy intake compared with placebo in healthy volunteers. Moreover, we hypothesized that post-oral delivery of non-caloric tastants results in increased satiation and the release of GI peptides as the primary mechanism of action. A meta-analysis was performed in case at least two studies described the use of non-caloric tastants of the same taste on the primary outcome energy intake and clinical heterogeneity was acceptable.

## Methods

### Search strategy

This systematic review and meta-analysis was registered in the PROSPERO database on 26 February 2020 (ID: CRD42020171182). The present systematic review and meta-analysis were performed according to the Preferred Reporting Items for Systematic Reviews and Meta-Analyses (PRISMA) guidelines [46]. The description of the PICOS (participants, intervention, comparison, outcome, and setting) criteria used to define the research question are depicted in Table 1. A structured search in the Cochrane, PubMed, Embase, and Medline databases was performed up to 26 February 2020 with the following search strategy: ((((((((((((tastant) OR Taste) OR Taste receptor) OR bitter taste) OR quinine) OR denatonium benzoate) OR umami) OR sodium glutamate) OR monosodium glutamate) OR sweet) OR non-nutritive sweeteners)) AND ((((((((((((((((energy intake) OR intake) OR food intake) OR appetite sensation) OR satiation) OR satiety response) OR satiety) OR satiety hormones) OR glucagon-like peptide-1) OR peptide YY) OR Ghrelin) OR leptin) OR cholecystokinin) OR motilin) OR motility) OR gastric emptying).

**Table 1:** Description of the PICOS criteria used to define the research question

Parameter	Description
Participants	Healthy individuals
Intervention	Prototypical non-caloric tastants at least once
Comparison	Prototypical non-caloric tastants vs. placebo
Outcomes	Energy intake, GI symptoms and perceptions, and mechanisms of effect
Setting	Randomized controlled trials with a parallel or crossover design
Research question	What is the effect of post-oral delivery of non-caloric tastants on energy intake in healthy volunteers? Secondary: what is the effect of post-oral delivery of non-caloric tastants on GI symptoms and perceptions and what is the effect of post-oral delivery of non-caloric tastants on mechanisms of action in healthy volunteers

### Selection criteria

Eligibility of each paper was assessed independently by two researchers (TK and DK) according to predefined criteria. Papers reporting the effects of post-oral delivery of non-caloric tastants on eating behavior (e.g., energy intake, GI peptides, appetite sensations, GI motility, GI symptoms, brain signaling and other effects) were included. To investigate the function of gastrointestinal taste receptors, papers needed to properly bypass oral taste effects. Therefore, studies investigating delivery methods using catheters, capsules or other methods resulting in adequate masking of oral taste were included. Moreover, papers were excluded if they were reviews, comments, replies on an original paper, or abstracts without available full text. No limitations on publication date were set. Paper inclusion was agreed upon by both reviewers. A third reviewer (AM) was consulted with regard to inclusion in case of disagreement between the two reviewers.

### Outcome measures

This systematic review looked at various aspects of eating behavior after post-oral delivery of non-caloric tastants. Outcome measures of interest were (1) energy intake, (2) GI symptoms and perceptions, and (3) mechanisms of effect.

### Data extraction

Two reviewers (TK) and (DK) carried out the data extraction. Two authors were contacted to elaborate their data and they replied. Name of author, year of publication, country, sample size, age of subjects, BMI of subjects, tastants used, method of administration, energy intake, appetite sensations, GI symptoms, GI peptides, GI motility, and brain signaling in homeostatic and hedonic regions were abstracted and presented in tables. Principle summary measures are differences in means.

### Quality assessment

Two independent reviewers (TK and DK) used the revised Cochrane risk of bias tool to assess risk of bias in randomized trials (RoB 2) to assess the quality of included papers [47]. The quality of the paper was assessed only once when a paper described multiple studies. A third reviewer (AM) was consulted in case of discordance between the two reviewers. The RoB 2 tool, assessing the quality of randomized controlled trials, consists of five domains covering bias arising from the randomization process, bias due to deviations from intended interventions, bias due to missing outcome data, bias in the measurement of the outcome, and bias in selection of the reported result. The scoring system assesses the risk of bias on these domains (low risk of bias, some concerns, high risk of bias). When an individual domain received score of a particular level of bias, overall risk of bias was determined to be at least as severe.

### Statistical analysis

A meta-analysis was performed if at least two studies described the use of non-caloric tastants of the same basic taste on energy intake and clinical heterogeneity between studies was acceptable. Clinical heterogeneity was discussed by two independent reviewers (TK and DK). In case of discordance between these reviewers, a third reviewer (AM) was consulted. Meta-analyses were performed using a random effect model by the metaphor package in R (version 3.6.3) [48]. Energy intake in Kcal after tastants and control were pooled using the data provided by included studies. Sensitivity analyses were performed when same studies employed different doses of tastants in order for those subjects to not influence the results to a greater extent than subjects form other studies. The *I*^2^ was used to quantitatively measure statistical heterogeneity between studies (*p* value < 0.05).

### Data reporting

**Fig. 1:**
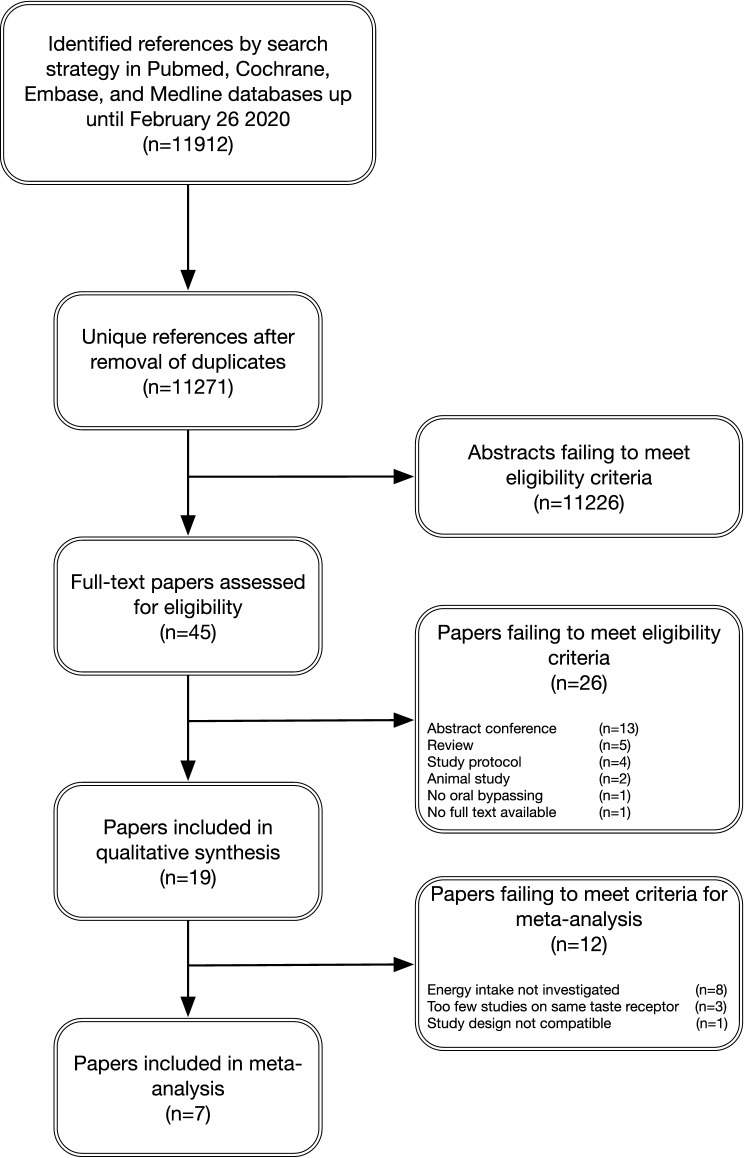
Flow chart of the selection process; from identification of possible eligible papers to papers included in this review and meta-analysis

Paper inclusion, exclusion, and reasons for exclusion are presented in a diagram (Fig. 1) according to the PRISMA statement for reporting a systematic review and meta-analysis.

## Results

### Systematic approach to paper selection

After removal of duplicates, a total of 11,271 abstracts were assessed, and 45 full texts were screened for eligibility (Fig. 1). A total of 19 papers, describing 25 studies met the inclusion criteria. Twenty-six papers were excluded for various reasons: abstract for a conference (13), review (5), study protocol (4), animal study (2), no adequate bypassing of oral taste receptors (1), no full text available (1). The flow-chart for screening and inclusion of papers is depicted in Fig. 1.

Supplementary Table 1 provides an overview of the results of the included papers.

### Quality assessment

**Fig. 2:**
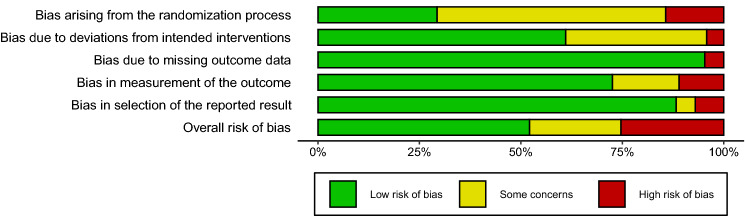
Risk of bias summary. Author’s judgements broken down for the domains according to the revised tool to assess risk of bias in randomized trials (RoB 2)

The results of the quality assessment are summarized in Fig. 2. Overall, papers scored decently on the risk of bias assessment. Bias arising from the randomization process was determined to raise some concerns by the majority of papers due to lacking information on the method of randomization. Most papers described randomization of subjects but provided no information on the method of randomization.

### Study characteristics

#### Study population

The characteristics of the 25 studies described in 19 papers are summarized in Supplementary Table 1. All studies included healthy volunteers [27–45]. Most studies included volunteers with a normal BMI between 18 and 25 kg/m^2^ [27–38, 40–42, 44, 45]. One study described inclusion of both subjects with a BMI between 18 and 25 kg/m^2^ and subjects with a BMI greater than 30 kg/m^2^ [43]. One study described inclusion of subjects with a BMI between 23 and 32 kg/m^2^ [39]. Eleven studies described inclusion of both women and men [27, 28, 34–38, 40–43], four studies described inclusion of men only [29, 30, 44, 45], and four studies described inclusion of women only [31–33, 39].

#### Tastants used

Table 2 provides an overview of the tastants used by included studies and the ligand receptors that are activated per tastant.

**Table 2:** Ligand receptors of the tastants described in the included studies. Ligand receptors for TAS2Rs adapted from BitterDB [49]

Tastant	Ligand receptors
Aspartame	TAS1R2/TAS1R3 heterodimer
Saccharin	TAS1R2/TAS1R3 heterodimer, TAS2R43, TAS2R44
Sucralose	TAS1R2/TAS1R3 heterodimer, TAS2R1, TAS2R4, TAS2R5, TAS2R7, TAS2R8, TAS2R10, TAS2R39, TAS2R41, TAS2R41, TAS2R46
Ace-K	TAS1R2/TAS1R3 heterodimer, TAS2R43, TAS2R44
Reb-A	TAS1R2/TAS1R3 heterodimer, TAS2R4, TAS2R14
Xylitol	TAS1R2/TAS1R3 heterodimer
Erythritol	TAS1R2/TAS1R3 heterodimer
Naringin	N/A
Quinine	TAS2R4, TAS2R7, TAS2R10, TAS2R14, TAS2R39, TAS2R40, TAS2R43, TAS2R44, TAS2R46
QHCl	TAS2R4, TAS2R7, TAS2R10, TAS2R14, TAS2R39, TAS2R40, TAS2R43, TAS2R44, TAS2R46
DB	TAS2R4, TAS2R8, TAS2R10, TAS2R13, TAS2R39, TAS2R43, TAS2R46, TAS2R47
Bitter secoiridoids (*Gentiana lutea* extract, contains amarogentin)	TAS2R1, TAS2R4, TAS2R39, TAS2R43, TAS2R46, TAS2R47, TAS2R50
Raisin flavor	N/A
Sucrose octaacetate	TAS2R46
Quassia extract	TAS2R4, TAS2R10, TAS2R14, TAS2R46, TAS2R47
Amarasate extract	N/A
MSG	TAS1R1/TAS1R3 heterodimer

*TAS1R* taste receptor 1, *TAS2R* taste receptor, *2Ace-K* acesulfame potassium, *Reb-A* rebaudioside A, *N/A* no data available, *QHCL* quinine hydrochloride, *DB* denatonium benzoate, *MSG* monosodium glutamate

#### Sweet tastants

Nine studies (reported on in eight papers) investigated post-oral delivery of sweet tastants. Aspartame alone was used in three studies (two papers) [40, 44], one study used aspartame and saccharin [35], one study used aspartame, acesulfame potassium (Ace-K), and sucralose [41], one study used sucralose [36], one study used rebaudioside A (Reb-A) [42], one study used xylitol and erythritol [43], and one study used only Ace-K [38]. It should be noted that Reb-A, Ace-K, Saccharin, and sucralose are known to activate bitter taste receptors aside from sweet taste receptors. TAS2R4 and 14 are activated by Reb-A, TAS2R43 and 44 are activated by Ace-K, TAS2R43 and 44 are activated by saccharin, and TAS2R1, 4, 5, 7, 8, 10, 39, 41, 46 are activated by sucralose [49]. However, given their predominant sweet taste [50–52] and their main role as non-nutritive sweeteners, for this paper, they were described as sweet tastants.

#### Bitter tastants

Sixteen studies (12 papers) investigated post-oral taste delivery of bitter tastants. Six of these studies used quinine alone [27, 29–31, 33, 42], five studies (two papers) used denatonium benzoate (DB) alone [28, 32], one study investigated quinine and naringin [35], one study used a bitter mixture consisting of raisin flavor, sucrose octaacetate, and quassia extract [39], one study used bitter secoiridoids [37], and one study used bitter New Zealand hop extracts [45]. Different bitter compounds activate different (combinations of) TAS2 receptor subtypes in humans [49, 53]. However, most extensively investigated are quinine and denatonium benzoate, which both activate four of the same TAS2 receptor subtypes, among other subtypes. DB activates eight TAS2 receptor subtypes in humans (TAS2R 4, 8, 10, 13, 39, 43, 46, and 47), whereas quinine activates nine subtypes of TAS2 receptor in humans (TAS2R 4, 7, 10, 14, 39, 40, 43, 44, and 46) [49, 53].

#### Umami tastants

Only one study investigated post-oral delivery of an umami tastant. Monosodium glutamate was used in this study [42].

#### Combination of tastants

Two studies investigated post-oral delivery of a combination of tastants (sweet, bitter, and umami). Both of these studies used quinine, Reb-A, and monosodium glutamate [34, 42].

#### Comparators

Most studies describe the use of a placebo. For studies using nasogastric, nasoduodenal or naso-duodenal-ileal delivery of tastants either tap water [32, 34, 35, 38, 41–43], saline [28–30, 36], or milli-Q water [31, 33] was used as a comparator. Most studies that used capsules to deliver the tastants used placebo capsules as a comparator [27, 39, 40, 45]. One study used a capsule to deliver tastants and used water without a capsule as comparator [44]. One study added microencapsulated bitter taste to a pudding and used only the coating as a comparator [37].

#### Energy intake

An overview of the studies describing effects of post-oral delivery of non-caloric tastants on energy intake is provided in Table 3.

**Table 3:** Studies describing the effects of post-oral delivery of non-caloric tastants on energy intake

Taste	References	Subjects	Tastants and comparators used	Method of administration	Interval intervention to meal	Energy intake (Kcal)	Direction of effect
Sweet	Rogers et al. (1990) [40] UK	12 subjects (6 men, 6 women, 18–26 y, BMI 20.8)	Aspartame capsule (234 mg) Comparator: Placebo capsule	Gastric capsule	60 min	− 175 kcal	↓
		15 subjects (10 men, 5 women, 19-24 y, normal BMI	Aspartame capsule (235 mg) Aspartame capsule (470 mg) Comparator: Placebo capsule	Gastric capsule	60 min	− 138 kcal for 235 mg aspartame − 150 kcal for 470 mg aspartame	↓
	Black et al. (1993) [44] Canada	18 subjects (18 men, 19-25y, BMI 21–25)	Aspartame capsule (340 mg) Comparator: Water	Gastric capsule	60 min	Slight non-significant increase in energy intake (numbers not shown) No effect on macronutrient composition	No effect
	Van Avesaat et al. (2015) [42] The Netherlands	15 subjects (6 men, 9 women, 22.4 y, BMI 22.4)	Reb-A (540 mg) Comparator: Tap water	Nasoduodenal catheter	75 min	− 24 kcal (n.s.)	No effect
Bitter	Andreozzi et al. (2015) [27] Italy	20 subjects (8 men, 12 women, 27 y, BMI 24)	QHCl capsule (18 mg) Comparator: Placebo capsule	Acid resistant capsules	60 min	-82 kcal	↓
	Van Avesaat et al. (2015) [42] The Netherlands	15 subjects (6 men, 9 women, 22.4 y, BMI 22.4)	QHCl (75 mg) Comparator: Tap water	Nasoduodenal catheter	75 min	− 44 kcal (n.s.)	No effect
	Mennella et al. (2016) [37] Italy	20 subjects (11 men, 9 women, 25.3 y, BMI 22.1	Microencapsulated bitter secoiridoids (100 mg) Comparator: coating only	Microencapsulation to mask oral tasting. Exact location of effect in GI tract unknown	180 min (lunch) 24 h energy intake	Lunch:—88 kcal (n.s.) Post-lunch: − 252 kcal 24 h energy intake: − 340 kcal	↓
	Peters et al. (2016) [39] The Netherlands	57 subjects (all women, 40.5 y, BMI 26.5)	Bitter mixture containing: Raisin flavor (22.0 mg) Sucrose Octa Acetate (0.88 mg) Quassia extract (0.088 mg) Comparator: placebo capsule	Intragastric capsule, 2 times daily for 14 days	60 min (breakfast) 300 min (lunch) 60 min (dinner) All day energy intake	Day 0 vs. day 14: Meals only: − 109 kcal (n.s.) Meals + snack: -86 kcal (n.s.) Breakfast: − 30 kcal (n.s.) Lunch: − 61 kcal (n.s.) Dinner: − 1 kcal (n.s.) Snacks: + 41 kcal (n.s.)	No effect
	Deloose et al. (2017) [32] Belgium	20 subjects (all women, 23 y, BMI 22)	DB (0.447 mg/Kg body weight) Comparator: Tap water	Nasogastric catheter	40 min	− 76 kcal (n.s.)	No effect
	Bitarafan et al. (2019) [30] Australia	14 subjects (14 men, 25 y, BMI 22.5)	QHCl (37.5 mg, Q37.5)) QHCl (75 mg, Q75)) QHCl (225 mg, Q225)) Comparator: Saline	Nasoduodenal catheter	60 min	Q37.5:—31Kcal (n.s.), Q75: − 59 kcal (n.s.), Q225: − 11 kcal vs. Control (n.s.)	No effect
	Iven et al. (2019) [33] Belgium	16 subjects (16 women, 24.5 y, BMI 21.9)	QHCl (3.6 mg/Kg body weigh) Comparator: Milli-Q water	Nasogastric catheter	40 min	− 67.6 kcal	↓
	Bitarafan et al. (2020) [29] Australia	12 subjects (12 men, 26 y, BMI 23.1)	QHCl (275 mg, Q275) QHCl (600 mg, Q600) Comparator: Saline	Nasogastric catheter	30 min	Q275: + 26 kcal, Q600: − 53 kcal (n.s.)	No effect
Umami	Van Avesaat et al. (2015) [42] The Netherlands	15 subjects (6 men, 9 women, 22.4 y, BMI 22.4)	MSG (2 g) Comparator: Tap water	Intraduodenal catheter	75 min	+ 5 kcal (n.s.)	No effect
Combination	Van Avesaat et al. (2015) [42] The Netherlands	15 subjects (6 men, 9 women, 22.4 y, BMI 22.4)	Tastant mixture: Reb-A (540 mg) QHCl (75 mg) MSG (2 g) Comparator: Tap water	Nasoduodenal catheter	75 min	− 64 kcal	↓
	Klaassen et al. (2019) [34] The Netherlands	14 subjects (3 men, 11 women, 25.6 y, BMI 22.3)	Tastant mixture: Reb-A (540 mg) QHCl (75 mg) MSG (2 g) Comparator: Tap water	Naso-duodenal-ileal catheter	75 min	Duodenal + 16.7 kcal (n.s.), Ileal + 28.1 kcal (n.s.), Combined duodenal and ileal + 31.5 kcal (n.s.)	No effect

*y* years, *BMI* body mass index, *Reb-A* rebaudioside A, *n.s.* not significant, *QHCL* quinine hydrochloride, *GI* gastrointestinal, *DB* denatonium benzoate, *MSG* monosodium glutamate

#### Sweet tastants

Four studies (three papers) reported the effect of sweet tastants on energy intake [40, 42]. Two studies (one paper) showed a significant decrease of 138, 150, and 175 kcal (*p* < 0.05, *p* < 0.01, and *p* < 0.02, respectively) of an ad libitum buffet meal after intragastric delivery of aspartame in various concentrations compared with placebo [40]. However, another study showed no effect on energy intake or macronutrient preferences during an ad libitum buffet meal after intragastric delivery of aspartame [44]. In line with this, one study failed to demonstrate a difference between Reb-A and placebo on ad libitum food intake intraduodenal delivery [42].

The data reported in two papers were not described in sufficient detail to use for pooling [40, 44]. The authors of these papers were contacted. Both authors responded and declared that raw data were not available anymore, since the studies were performed over 30 years ago. Therefore, these studies could not be pooled.

#### Bitter tastants

Eight studies showed the effect of post-oral delivery of bitter tastants on energy intake. Three of these described a decrease in energy intake after intragastric [33], intraduodenal [27], or post-oral delivery of bitter tastants [37]. On the other hand, five studies showed no effect on energy intake after gastrointestinal delivery of bitter tastants. However, most of these studies described a modest decrease in energy intake that did not reach statistical significance [29, 30, 32, 39, 42].

Iven et al. showed a decrease of 67.7 kcal of hedonic eating after intragastric infusion of quinine compared with control [33]. Andreozzi et al. showed a decrease of 82 kcal after an acid resistant capsule containing quinine compared with a placebo capsule [27]. Mennella et al. showed no significant decrease in lunch intake (− 88 kcal) after microencapsulated bitter secoiridoids compared with control, but a significant decrease of post-lunch energy intake (− 252 kcal) and 24 h energy intake (− 340 kcal) [37].

Van Avesaat et al. showed a non-significant decrease of 44 kcal after intraduodenal infusion of quinine compared with control [42]. Peters et al. investigated energy intake after a 2-week, two times daily consumed capsule containing a bitter mixture compared with control [39]. They showed a non-significant decrease of daily meal intake (− 109 kcal), daily meal intake including snacks (− 86 kcal), breakfast (-30 kcal), lunch (− 61 kcal), and dinner (− 1 kcal). A non-significant increase of 41 kcal on snacks only was found [39]. Deloose et al. showed that intragastric infusion of DB resulted in a non-significant decrease of 76 kcal compared with control [32]. In one study, Bitarafan et al. showed a non-significant decrease in food intake following various doses of intraduodenally administered quinine compared with control (− 31 kcal for 37.5 mg, − 59 kcal for 75 mg, and − 11 kcal for 225 mg) [30]. In another study, Bitarafan et al. showed a non-significant increase in energy intake of 26 kcal after intragastric administration 275 mg of quinine, whereas intragastric administration of 600 mg quinine showed a non-significant decrease of 53 kcal [29].

**Fig. 3:**
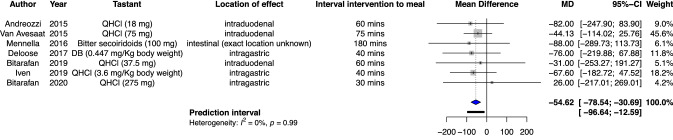
Forest plot for pooled mean difference in energy intake after bitter components versus placebo. For the papers Bitarafan et al. [30] and Bitarafan et al. the lowest dose is depicted. *QHCl* quinine hydrochloride, *DB* denatonium benzoate

Seven studies investigating the effect of gastrointestinal delivery of bitter tastants on energy intake could be pooled [27, 29, 30, 32, 33, 37, 42] and are depicted in Fig. 3. One study looked at total energy intake during the day as well as energy intake during the lunch [37]. Pooled effects were calculated with the outcome of lunch intake for this study to minimize clinical heterogeneity. One study investigating the effect of intragastric bitter tastant delivery on energy intake could not be pooled because the design employed in that study differed too much from the other designs [39]. Two studies employed various doses of QHCl in the same population [29, 30]. In order for those subjects to not influence the results to a greater extent than subjects form other studies, the meta-analysis was performed for all the combinations of doses. The lowest dose for both studies is depicted in Fig. 3. Pooled effects show a significant reduction in caloric intake of 54.62 kcal (95% CI − 78.54 − 30.69, *p* = 0.0014). A sensitivity analysis was performed for all the combinations of doses employed by Bitarafan et al. and results are depicted in Supplementary Fig. 1. Decrease in caloric intake varies between 53 and 58 kcal for the different combinations, all statistically significant [29, 30].

#### Umami tastants

Van Avesaat et al. showed no effect on energy intake after intraduodenal delivery of monosodium glutamate compared with placebo [42].

#### Combination of tastants

Van Avesaat et al. showed a significant decrease of energy intake of 64 kcal after intraduodenal delivery of a combination of quinine, Reb-A and monosodium glutamate. A study conducted by the same research group showed no effects on energy intake after intraduodenal and/or intraileal delivery of the same tastant mixture [42]. These studies were not pooled, due to high clinical heterogeneity [34].

### GI symptoms and perceptions

An overview of the studies describing effects of post-oral delivery of non-caloric tastants on GI symptoms and perceptions is provided in Table 4.

**Table 4:** Studies describing the effects of post-oral delivery of non-caloric tastants on GI symptoms and perceptions

Taste	References	Subjects	Tastants and comparators used	Method of administration	GI symptoms and perceptions	Direction of effect
Sweet	Rogers et al. (1990) [40] UK	15 subjects (10 men, 5 women, 19-24 y, normal BMI	Aspartame capsule (235 mg) Aspartame capsule (470 mg) Comparator: Placebo capsule	Gastric capsule	Aspartame capsules reduced desire to eat and hunger scores Aspartame capsules tended to increase fullness compared with placebo (n.s.)	↓ desire to eat/hunger No effects fullness
	Black et al. (1993) [44] Canada	18 subjects (18 men, 19–25 y, BMI 21–25)	Aspartame capsule (340 mg) Comparator: Water	Gastric capsule	No effects of aspartame on appetite sensations	No effects
	Little et al. (2009) [35] UK	10 subjects	Saccharin (50 mg) Aspartame (200 mg) Comparator: Tap water	Nasogastric catheter	No effects of aspartame or saccharin on hunger or fullness	No effects
	Steinert et al. (2011) [41] Switzerland	12 subjects (6 men, 6 women, 23.3 y, BMI 23.0)	Aspartame (160 mg) Ace-K (200 mg) sucralose (62 mg) Comparator: Tap water	Nasogastric catheter	Artificial sweeteners reduced hunger, and increased satiety and fullness ratings to an intermediate amount between water and carbohydrate sugars (n.s.)	No effects
	Van Avesaat et al. (2015) [42] The Netherlands	15 subjects (6 men, 9 women, 22.4 y, BMI 22.4)	Reb-A (540 mg) Comparator: Tap water	Nasoduodenal catheter	Reb-A did not influence appetite sensations. Reb-A did not induce GI symptoms	No effects
	Wölnerhanssen et al. (2016) [43] Switzerland	20 subjects 10 lean subjects (5 men, 5 women, 26.6 y, BMI 21.7) 10 obese subjects (5 men, 5 women, 27.2 y, BMI 40.0)	Xylitol (50 g) Erythritol (75 g) Comparator: Tap water	Nasogastric catheter	Both sweeteners did not affect appetite sensations. Xylitol and erythritol led to bloating and diarrhea in 70% and 60% of subjects, respectively	No effects appetite sensations ↑Side effects
	Meyer-Gerspach et al. (2018) [38] Belgium	12 subjects (6 men, 6 women, 23 y, BMI 23)	Ace-K (220 mg) Comparator: Tap water	Nasogastric catheter	Hunger: Strong initial decrease in hunger after Ace-K with a faster return of hunger after first time point and slower return of hunger in last part of curve after Ace-K vs. Control Satiation: Strong initial increase in satiation after Ace-K vs. control with faster decrease after first time point and slower decrease in last part of curve after Ace-K vs. control No adverse events	↓Hunger ↑Satiation No adverse events
Bitter	Little et al. (2009) [35] UK	12 subjects	Naringin (290.27 mg) Quinine (32.2 mg) Comparator: Tap water	Nasogastric catheter	No effects of naringin or quinine on appetite sensations	No effects
	Andreozzi et al. (2015) [27] Italy	20 subjects (8 men, 12 women, 27 y, BMI 24)	QHCl capsule (18 mg) Comparator: Placebo capsule	Acid resistant capsules	QHCl did not affect satiety or desire to eat scores vs. Control No adverse events	No effects No adverse events
	Avau et al. (2015) [28] Belgium	12 subjects (5 men, 30.6 y, BMI 23.8)	DB (0.447 mg/kg body weight) Comparator: Saline	Nasogastric catheter	DB made subjects feel satiated earlier and at lower volumes during constant nutrient infusion No adverse effects	↑Satiation No adverse events
	Van Avesaat et al. (2015) [42] The Netherlands	15 subjects (6 men, 9 women, 22.4 y, BMI 22.4)	QHCl (75 mg) Comparator: Tap water	Nasoduodenal catheter	Quinine did not influence appetite sensations Quinine did not induce GI symptoms	No effects No GI symptoms
	Mennella et al. (2016) [37] Italy	20 subjects (11 men, 9 women, 25.3 y, BMI 22.1	Bitter secoiridoids (100 mg) Comparator: Coating only	Microencapsulation to mask oral tasting. Exact location of effect in GI tract unknown	No effect of bitter encapsulate on fullness, satiety, hunger or desire to eat	No effects
	Deloose et al. (2017) [32] Belgium	20 subjects (10 men, 10 women, 27 y, BMI 24)	DB (0.447 mg /Kg body weight) Comparator: Tap water	Nasogastric catheter	Women: Switch from gastric to duodenal phase 3 origin was accompanied by lower percentage change of hunger scores after DB vs. Control Men: Percentage change in hunger scores during phase 3 contraction did not differ after DB vs. Control (n.s.) No adverse events after DB administration	↓Hunger in women
		12 subjects (all women, 31 y, BMI 22)	DB (0.447 mg /Kg body weight) Comparator: Tap water	Nasogastric catheter	No adverse events after DB administration	No adverse events
		13 subjects (all women, 28 y, BMI 23)	DB (0.447 mg /Kg body weight) Comparator: Tap water	Nasogastric catheter	Hunger scores after a standardized meal were lower after DB vs. Control. Satiety scores were higher after a standardized meal after DB No adverse events after DB administration	↓Hunger ↑Satiety No adverse events
		20 subjects (all women, 23 y, BMI 22)	DB (0.447 mg /Kg body weight) Comparator: Tap water	Nasogastric catheter	No adverse events after DB administration	No adverse events
	Deloose et al. (2018) [31] Belgium	10 subjects (10 women, 33 y, BMI 22)	QHCl (3.6 mg/kg body weight) Comparator: Milli-Q water	Nasogastric catheter	No adverse events	No adverse events
	Bitarafan et al. (2019) [30] Australia	14 subjects (14 men, 25 y, BMI 22.5)	QHCl (37.5 mg, Q37.5)) QHCl (75 mg, Q75)) QHCl (225 mg, Q225)) Comparator: Saline	Nasoduodenal catheter	No differences in VAS scores for hunger, desire to eat, prospective consumption, or fullness after Q37.5, Q75, or Q225 vs. Control No adverse events, no effects of Q37.5, Q75, or Q225 on nausea or bloating	No effects No GI symptoms No adverse events
	Iven et al. (2019) [33] Belgium	16 subjects (16 women, 24.5 y, BMI 21.9)	QHCl (3.6 mg/Kg body weigh) Comparator: Milli-Q water	Nasogastric catheter	Hunger scores increased after control and decreased after QHCl (n.s.) Prospective food consumption scores decreased after QHCl vs. Control Satiety scores increased after QHCl vs. Control Fullness scores increased after QHCl vs. control Minimal nausea scores reported (n.s.)	↓Prospective food consumption ↑Satiety ↑Fullness No GI symptoms
	Walker et al. (2019) [45] New Zealand	30 subjects (30 men, 24y, BMI 23.1)	Amarasate extract (500 mg, HD) Amarasate extract (200 mg, LD) Comparator: Placebo capsule	Acid resistant capsule	From T = 90 onwards HD and LD show lower mean changes in hunger and fullness Lower mean changes in fullness for HD from t = 120 onwards, only t = 180 and t = 330 for LD No nausea. 3 participants in HD and 1 in LD had liquid loose bowel movements	↓Hunger ↑Fullness No GI symptoms
	Bitarafan et al. (2020) [29] Australia	15 subjects (15 men, 26 y, BMI 23.2)	QHCl (275 mg, Q275) Quinine-HCl (600 mg, Q600) Comparator: Saline	Nasogastric catheter	No effects of Q275 or Q600 on hunger, desire to eat, prospective consumption, or fullness scores No effects of Q275 or Q600 on bloating or nausea vs. Control. No other adverse effects	No effects No GI symptoms No adverse events
		12 subjects (12 men, 26 y, BMI 23.1)	QHCl (275 mg, Q275) QHCl (600 mg, Q600) Comparator: Saline	Nasogastric catheter	No effects of Q275 or Q600 on hunger, desire to eat, prospective consumption, or fullness scores No effects of Q275 or Q600 on bloating or nausea vs. Control. No other adverse effects	No effects No GI symptoms No adverse events
Umami	Van Avesaat et al. (2015) [42] The Netherlands	15 subjects (6 men, 9 women, 22.4 y, BMI 22.4)	MSG (2 g) Comparator: Tap water	Intraduodenal catheter	MSG decreased hunger and desire to eat but did not influence satiation or fullness MSG did not induce GI symptoms	↓ Desire to eat/hunger No effects satiation/fullness No GI symptoms
Combination	Van Avesaat et al. (2015) [42] The Netherlands	15 subjects (6 men, 9 women, 22.4 y, BMI 22.4)	Tastant mixture: Reb-A (540 mg) QHCl (75 mg) MSG (2 g) Comparator: Tap water	Nasoduodenal catheter	The tastant mixture decreased hunger and desire to eat, but not satiation or fullness The tastant mixture did not induce GI symptoms	↓ Desire to eat/hunger No effects satiation/fullness No GI symptoms
	Klaassen et al. (2019) [34] The Netherlands	14 subjects (3 men, 11 women, 25.6 y, BMI 22.3)	Tastant mixture: Reb-A (540 mg) QHCl (75 mg) MSG (2 g) Comparator: Tap water	Naso-duodenal-ileal catheter	No effects of duodenal-, ileal- or combined duodenal and ileal delivery of non-caloric tastants on appetite sensations The tastant mixture did not induce GI symptoms	No effects No GI symptoms

*y* years, *BMI* body mass index, *n.s.* not significant, *Ace-K* acesulfame potassium, *Reb-A* rebaudioside A, *QHCL* quinine hydrochloride, *DB* denatonium benzoate, *GI* gastrointestinal, *MSG* monosodium glutamate

#### Sweet tastants

Seven studies investigated the effects of gastrointestinal delivery of sweet tastants on appetite sensations. Five of these studies showed no effects on appetite sensations [35, 41–44]. One study showed that intragastric delivery of aspartame reduced desire to eat without influencing fullness [40]. Another study demonstrated a strong initial decrease in hunger and increase in satiety, with faster returns to baseline after intragastric delivery of Ace-K compared with control [38].

Three studies examined the effects of post-oral administration of sweet tastants on GI symptoms and other adverse events [38, 42, 43]. Wölnerhanssen et al. showed that intragastric administration of xylitol and erythritol leads to bloating and diarrhea in 70% and 60% of subjects, respectively [43]. Other studies reported no GI symptoms or adverse events [38, 42].

#### Bitter tastants

Five studies (four papers) showed that post-oral delivery of bitter tastants resulted in a decrease of hunger and prospective food consumption and an increase of satiation/satiety and fullness [28, 32, 33, 45]. However, six studies showed no effects on appetite sensations after post-oral delivery of bitter tastants [27, 29, 30, 35, 37, 42].

Seven studies examined the effects of post-oral delivery of bitter tastants on GI symptoms and/or adverse events. None of these reported side effects or adverse events [27, 29–33, 42].

#### Umami tastants

One study described that post-oral delivery of umami decreased the desire to eat and hunger, without influencing satiation, fullness or GI symptoms [42].

#### Combination of tastants

Two studies described the effects of post-oral delivery of a combination of sweet, bitter and umami tastants on appetite sensations. One study described a decrease of desire to eat and hunger, whereas satiation and fullness were not attenuated [42]. The other study showed no effects on appetite sensations [34].

No GI symptoms or adverse events after post-oral delivery of this combination of sweet, bitter, and umami tastants were reported [34, 42].

### Mechanisms of effect

An overview of the studies describing effects of post-oral delivery of non-caloric tastants on the mechanisms of effect involved in regulating eating behavior is provided in Table 5. The mechanisms of interest were GI peptide release, GI motility, and brain signaling.

**Table 5:** Studies describing the effects of post-oral delivery of non-caloric tastants on the mechanisms of effects that influence eating behavior such as GI peptides, GI motility and brain signaling

Taste	References	Subjects	Tastants and comparators used	Method of administration	Mechanisms of effect	Direction of effect
Sweet	Little et al. (2009) [35] UK	10 subjects	Saccharin (50 mg) Aspartame (200 mg) Comparator: Tap water	Nasogastric catheter	No effects of aspartame or saccharin on GE	No effects GE
	Ma et al. (2009) [36] Australia	7 subjects (24 y, BMI 21.6)	Sucralose (80 mg) Sucralose (800 mg) Comparator: Saline	Nasogastric catheter	No effects of sucralose on GE, plasma glucose, plasma insulin, plasma GLP-1, or plasma GIP	No effects GE No effects GI peptides
	Steinert et al. (2011) [41] Switzerland	12 subjects (6 men, 6 women, 23.3 y, BMI 23.0)	Aspartame (160 mg) Ace-K (200 mg) Sucralose (62 mg) Comparator: Tap water	Nasogastric catheter	Sweeteners did not affect plasma GLP-1, PYY, ghrelin, glucose, insulin, or glucagon	No effects GI peptides
	Van Avesaat et al. (2015) [42] The Netherlands	15 subjects (6 men, 9 women, 22.4 y, BMI 22.4)	Reb-A (540 mg) Comparator: Tap water	Nasoduodenal catheter	Reb-A did not affect plasma CCK, GLP-1, or PYY	No effects GI peptides
	Wölnerhanssen et al. (2016) [43] Switzerland	20 subjects 10 lean subjects (5 men, 5 women, 26.6 y, BMI 21.7) 10 obese subjects (5 men, 5 women, 27.2 y, BMI 40.0)	Xylitol (50 g) Erythritol (75 g) Comparator: Tap water	Nasogastric catheter	Plasma CCK, plasma GLP-1, Plasma glucose increased after xylitol and erythritol vs. control Plasma insulin increased after xylitol, but not after erythritol vs. control Gastric emptying was slowed during the first 60 min after xylitol and erythritol vs. Control	↓GE ↑plasma CCK, GLP-1, Glucose, insulin
	Meyer-Gerspach et al. (2018) [38] Belgium	12 subjects (6 men, 6 women, 23 y, BMI 23)	Ace-K (220 mg) Comparator: Tap water	Nasogastric catheter	No effect of Ace-K on plasma motilin, octanoylated ghrelin, active GLP-1, CCK, gastrin, and glucose GI motility did not differ between Ace-K and control A faster linear decrease in IGP from first post infusion time point, quicker return of IGP and quicker flattening of the curve during IGP recovery after Ace-K vs. Control with faster return to baseline in last part of the IGP curve after Ace-K vs. control	No effects GI motility ↓ IGP No effects GI peptides
Bitter	Little et al. (2009) [35] UK	12 subjects	Naringin (290.27 mg) Quinine (32.2 mg) Comparator: Tap water	Nasogastric catheter	No effects of naringin or quinine on gastric emptying compared with water	No effects on GE
	Andreozzi et al. (2015) [27] Italy	20 subjects (8 men, 12 women, 27 y, BMI 24)	QHCl capsule (18 mg) Comparator: Placebo capsule	Acid resistant capsules	CCK: Higher ΔT90 vs T0 and ΔT90 vs T60 after QHCl vs. Control GE (evaluated in 8 subjects): no differences in GE between QHCl (87 min) vs. Control (88 min)	No effect on GE ↑ CCK
	Avau et al. (2015) [28] Belgium	12 subjects (5 men, 30.6 y, BMI 23.8)	DB (0.447 mg/kg body weight) Comparator: Saline	Nasogastric catheter	Less drop in IGP after DB	↑ IGP
	Van Avesaat et al. (2015) [42] The Netherlands	15 subjects (6 men, 9 women, 22.4 y, BMI 22.4)	QHCl (75 mg) Comparator: Tap water	Nasoduodenal catheter	Quinine did not affect plasma CCK, GLP-1, or PYY levels	No effects on GI peptides
	Mennella et al. (2016) [37] Italy	20 subjects (11 men, 9 women, 25.3 y, BMI 22.1	Bitter secoiridoids (100 mg) Comparator: Coating only	Microencapsulation to mask oral tasting. Exact location of effect in GI tract unknown	Bitter encapsulate decreased plasma GLP-1 at 30 min, but had no effect on blood glucose, plasma amylin, plasma ghrelin, plasma glucagon, plasma GIP, plasma insulin, plasma leptin, plasma PP, or plasma PYY levels vs. Control	↑ GLP-1 at 30 min No effects on other GI peptides
	Deloose et al. (2017) [32] Belgium	20 subjects (10 men, 10 women, 27 y, BMI 24)	DB (0.447 mg/Kg body weight) Comparator: Tap water	Nasogastric catheter	Women: DB reduced number of gastric phase 3 contractions from 67% (control) to 33% (DB) in women Interval between IG administration and occurrence of phase 3 did not differ between control (76 min) and DB (93 min) in women (n.s.) Men: No difference in origin of phase 3 contractions between control (57% gastric) and DB (40% gastric) in men (n.s.) Interval between IG administration and occurrence of phase 3 did not differ between control (76 min) and DB (111 min) in men (n.s.)	↓ Gastric phase 3 contractions in women
		12 subjects (all women, 31 y, BMI 22)	DB (0.447 mg/Kg body weight) Comparator: Tap water	Nasogastric catheter	Plasma motilin was lower after DB vs. Control. No differences between plasma total ghrelin or octanoylated ghrelin after DB vs. Control	↓Plasma motilin No effect ghrelin
		13 subjects (all women, 28 y, BMI 23)	DB (0.447 mg/Kg body weight) Comparator: Tap water	Nasogastric catheter	GE (measured in 6 subjects) did not differ between control and DB (both 109 min	No effects on GE
	Deloose et al. (2018) [31] Belgium	10 subjects (10 women, 33 y, BMI 22)	QHCl (0.447 mg/kg body weight) Comparator: Milli-Q water	Nasogastric catheter	Plasma motilin and plasma ghrelin levels decreased after QHCl. No difference in plasma octanoylated ghrelin levels Time* treatment effect for antral motility. No main effect of treatment No effects of QHCl on duodenal motility	↓Antral motility ↓ plasma motilin and ghrelin
	Bitarafan et al. (2019) [30] Australia	14 subjects (14 men, 25 y, BMI 22.5)	QHCl (37.5 mg, Q37.5)) QHCl (75 mg, Q75)) QHCl (225 mg, Q225)) Comparator: Saline	Nasoduodenal catheter	No effects of Q37.5, Q75, and Q225 on plasma CCK or blood glucose vs. Control No effect of Q37.5, Q75, or Q225 on antral pressure waves, basal pyloric pressure, isolated pyloric pressure waves, and duodenal pressure waves vs. Control	No effects on GI motility No effects on GI peptides
	Iven et al. (2019) [33] Belgium	16 subjects (16 women, 24.5 y, BMI 21.9)	QHCl (3.6 mg/Kg body weigh) Comparator: Milli-Q water	Nasogastric catheter	Decreases in total ghrelin, octanoylated ghrelin, and motilin after QHCl vs. control Brain activity in homeostatic and hedonic regions: Increased activity after QHCl vs. Control in anterior insula, ACC, amygdala, putamen, nucleus accumbens, pallidum, caudate head and caudate body, medial and lateral OFC, hypothalamus and midbrain Decreased activity in brainstem/medulla	↓ plasma ghrelin and motilin ↑Activity in homeostatic and hedonic brain regions ↓ activity in brainstem/medulla
	Bitarafan et al. (2020) [29] Australia	15 subjects (15 men, 26 y, BMI 23.2)	QHCl (275 mg, Q275) QHCl (600 mg, Q600) Comparator: Saline	Nasogastric catheter	Plasma insulin was increased 30 min after Q275 and Q600 vs. Control No effects of Q275 or Q600 on plasma glucose, plasma glucagon, or plasma GLP-1 After mixed nutrient drink: Q275 and Q600 lowered glucose Q275 and Q600 increased plasma insulin No difference in glucagon response after nutrient drink Q275 increased plasma GLP-1, Q600 did not No effects of Q275 or Q600 on gastric emptying	No effects on GE ↑ plasma insulin after intervention alone ↓ glucose after nutrient drink ↑ plasma insulin after nutrient drink ↑ plasma GLP-1 after nutrient drink
Umami	Van Avesaat et al. (2015)[42] The Netherlands	15 subjects (6 men, 9 women, 22.4 y, BMI 22.4)	MSG (2 g) Comparator: Tap water	Intraduodenal catheter	MSG did not affect plasma CCK, GLP-1, or PYY levels	No effects on GI peptides
Combination	Van Avesaat et al. (2015) [42] The Netherlands	15 subjects (6 men, 9 women, 22.4 y, BMI 22.4)	Tastant mixture: Reb-A (540 mg) QHCl (75 mg) MSG (2 g) Comparator: Tap water	Nasoduodenal catheter	The tastant mixture did not affect plasma CCK, GLP-1, or PYY levels	No effects on GI peptides

*y* years, *BMI* body mass index, *GE* gastric emptying, *GLP-1* glucagon-like peptide 1, *GIP* glucose-dependent insulinotropic polypeptide, *GI* gastrointestinal, *Ace-K* acesulfame potassium, *PYY* peptide yy, *Reb-A* rebaudioside A, *CCK* cholecystokinin, *IGP* intragastric pressure, *QHCL* quinine hydrochloride, *DB* denatonium benzoate, *PP* pancreatic polypeptide, *IG* intragastric, n.s.: not significant, *ACC* anterior cingulate cortex, *OFC* orbitofrontal cortex, *MSG* monosodium glutamate

#### Sweet tastants

One study described a drop in intragastric pressure after intragastric administration of Ace-K [38]. Intragastric administration of xylitol and erythritol also resulted in slower gastric emptying [43]. However, another study showed no changes in gastric emptying after intragastric administration of saccharin or aspartame [35].

Five studies investigated the effects of post-oral delivery of sweet tastants on GI peptides [36, 38, 41–43]. Four of these showed no effects on GI peptide plasma levels [36, 38, 41, 42]. One study described that intragastric administration of xylitol and erythritol increases plasma CCK, GLP-1, and glucose [43].

#### Bitter tastants

Eight studies (seven papers) investigated the effects of post-oral delivery of bitter tastants on GI motility [27–32, 35]. Two of these showed a decrease in gastric phase 3 contractions after intragastric delivery of bitter tastants [31, 32]. One study described a relative increase in intragastric pressure after DB compared with placebo [28]. Four studies showed no effect of post-oral delivery of bitter tastants on gastric emptying [27, 29, 32, 35]. Another study showed no effect of intraduodenal delivery of quinine on antral, pyloric or duodenal pressure waves [30].

One study showed that intragastric delivery of quinine resulted in increased brain activity in homeostatic and hedonic brain regions [33].

Eight studies examined the effects of post-oral delivery of bitter tastants on GI peptides [27, 29–33, 37, 42]. Two studies showed that post-oral delivery of bitter tastants did not result in changes in GI peptides [30, 42]. One study showed an initial effect of quinine on plasma insulin, but not on plasma glucose, glucagon or GLP-1. However, a decrease of glucose and an increase of insulin was found after a standardized nutrient drink following quinine administration [29]. Another study showed a decrease in GLP-1 30 min after intervention but no effects on other GI peptides, nor an overall intervention effect on plasma GLP-1 levels [37]. Three studies showed a decrease in motilin and/or ghrelin after intragastric delivery of bitter tastants [31–33]. Only one study showed an increase of CCK after intraduodenal delivery of quinine [27].

#### Umami tastants

One study investigated the mechanisms of effect of post-oral delivery of monosodium glutamate, showing no changes in GI peptides (CCK, GLP-1, and PYY) after intraduodenal administration of MSG [42].

#### Combination of tastants

Only one study investigated the effect of post-oral delivery of a combination of tastants. No effect of intraduodenal administration of a combination of sweet, bitter and umami tastant mixture on plasma CCK, GLP-1, or PYY was found [42].

## Discussion

In this systematic review and meta-analysis, the currently available data on the effects post-oral delivery of non-caloric tastants has been evaluated. This review shows that the effects of post-oral delivery of non-caloric tastants on eating behavior are inconclusive and inconsistent thus far.

### Tastants: sweet, biter and umami

Most studies described the effects of post-oral delivery of sweet [35, 36, 38, 40–44] or bitter [27–33, 35, 37, 39, 42, 45] stimuli. Only one study used an umami stimulus [42] and two studies described the effects of a tastant mixture [34, 42]. It has been hypothesized that taste can predict the type of food that is being ingested (i.e., bitter for potential toxic substances, umami for glutamate and sweet for saccharides) [8], although this theory is probably an oversimplification and does not sufficiently reflect the complexity of the underlying biological responses. According to traditional beliefs, carbohydrates are considered as fuel for the human body [54], whereas one wants to avoid toxic substances [55]. Therefore, it is conceivable that most researchers hitherto have generally focused on sweet and bitter substances. However, umami taste is a predictor of amino acids. Up to now, little is known about solely post-oral effects of umami taste, although it has been widely accepted as a basic taste since the discovery of umami taste receptors in 2002 [56, 57]. However, more data are available on oral delivery of umami stimuli. Oral delivery of umami stimuli has been shown to elicit a GLP-1 response [58, 59]. Moreover, adding MSG to a novel flavor is able to condition liking for that flavor [60]. Furthermore, Dermiki et al. showed that adding MSG to novel flavored soups resulted in increased food intake in elderly subjects without eliciting increased liking [61]. Given the effects of oral MSG on food liking, food intake, and GI peptides, the effect of post-oral delivery of umami tastants should be further elucidated.

### Energy intake

This review and meta-analysis clearly shows that the most potent stimuli to influence eating behavior and reduce food intake are the bitter substances. The obvious explanation for this is the innate aversion for bitter taste [62]. That is, delivery of solely bitter tastants, in the absence of other flavors, would result in a warning signal with the intention to stop the intake of that particular substance. However, it should be noted that several studies show that this negative affective response to bitter can be decoupled by, for instance, the positive response to caffeine [9, 10]. This process is called flavor-consequence learning [63]. Moreover, Higgins et al. showed an increase in pale ale intake in individuals with increased bitter perception [64]. This indicates that, over the years, humans have learned to appreciate bitter tastes, mainly when combined with other flavors (*i.e.*, liking for black coffee and beers).

The question, however, arises whether any such mechanism would also hold true for post-oral taste receptor stimulation.

Data on post-oral delivery of sweet tastants are limited to three studies showing contradictory results [40, 42, 44]. Interestingly, more data are available on the effect of oral consumption of non-caloric sweeteners on energy intake. A review and meta-analysis by Rogers et al. that also included studies using oral stimulation showed a reduction of energy intake after consumption of low caloric sweeteners when compared with sugar consumption but not when compared to consumption of water in a short-term setting [65]. Since there is a preference for sweet foods and beverages in humans, further research to investigate the effects of post-oral delivery of sweet tastants on energy intake is warranted.

Eight studies described effects on energy intake after post-oral delivery of bitter tastants. These studies show a modest decrease in energy intake following post-oral delivery of bitter tastants [27, 29, 30, 32, 33, 37, 39, 42]. These decreases in energy intake varied from 11 to 88 kcal in the acute setting to 340 kcal on a daily basis. Pooled effects of post-oral delivery of bitter tastants show a significant decrease of 54 kcal compared with placebo in the acute setting. In itself, a reduction of 54 kcal in a single meal is rather small. However, in case this reduction in energy intake can be replicated several times daily over a longer period of time, this may indeed lead to a daily caloric deficit and subsequent weight loss. It should be noted, however, that these modest effects on caloric intake point towards a role in weight control rather than weight loss.

### GI symptoms and perceptions

Based on the included papers, no clear effects of post-oral delivery of non-caloric tastants on appetite sensations was found. However, it must be noted that appetite sensations are rarely measured as a primary outcome. Consequently, most studies might not have been adequately powered to detect differences in appetite sensations. Therefore, interpretation of these results should be done with care.

Interesting to point out is the finding of Deloose et al. showing a longer sustained satiation in response to bitter after a standardized test meal [32]. This observation indicates that adding a bitter tastant to a caloric carrier could result in a delay until the next meal. Such a combination could result in a decrease in snacking in between meals. In line with this, Mennella et al. found in their study a reduction in caloric consumption during the day, after intake of a breakfast containing an encapsulated bitter mixture leading to prolonged satiation [37].

Only one study investigated the effect of post-oral delivery of an umami stimulus. A decrease in desire to eat and hunger was found [42].

### Safety

According to the studies described in this review, noncaloric tastant administration is considered to be safe. One study reported side effects after administration of high doses of xylitol and erythritol, which was not surprising given the doses employed [43]. Other studies showed no GI-symptoms or other side effects after post-oral delivery of non-caloric tastants [27–34, 38, 42, 45]. It should be taken into account that, almost all studies focused on acute effects of post-oral delivery of non-caloric tastants and not on prolonged, daily administration.

The United States Food and Drug Administration (U.S. FDA) issues an acceptable daily intake (ADI) for various food components, including tastants. For example, the U.S. FDA issued a code of regulations stating soft drinks are allowed to contain 83 parts per million quinine [66]. Moreover, a systematic review describing the use of quinine to treat muscle cramps showed an increase of gastrointestinal complaints, headache and tinnitus after daily intake of 200–500 mg of quinine for 3 days up to several weeks [67]. Even more, the Medicines and Healthcare products Regulatory Agency (MHRA) issued a reminder on the dose-dependent effects on the QT interval [68]. Therefore, prolonged intake of high doses or combination with other drugs that prolong the QT interval should be avoided. Interestingly, in studies included in this review, the dose of the bitter tastant quinine ranged from 18 to 600 mg [27, 29–31, 33–35, 42]. This did not result in side effects in the studies investigating the acute effects of these compounds. However, when applying quinine daily to prevent obesity, the maximum dose should be carefully considered. This should be considered in future study protocols.

### Mechanisms of effect

Gastric emptying was delayed by high doses of xylitol and erythritol [43]. However, other studies showed no effect of post-oral delivery of non-caloric tastants on gastric emptying [27, 29, 32, 35]. Therefore, gastric emptying does not seem to be attenuated by post-oral delivery of non-caloric tastants.

GI motility appears to be attenuated as one research group has consistently found gastric motility and the GI peptides motilin and ghrelin to be affected by post-oral delivery of non-caloric tastants [28, 31–33]. Moreover, this research group recently published a review that elaborated the role of motilin as a regulator of hunger and food intake in humans [69], which indicates motilin as a possible target in combating the obesity epidemic. These interesting data are awaiting replication by other research groups.

Four studies showed increased CCK and/or GLP-1 levels after post-oral delivery of non-caloric tastants [27, 29, 37, 43]. Other studies that investigated these traditional satiety peptides did not find any effect of post-oral delivery of non-caloric tastants [30, 36, 38, 42]. This raises the question whether the focus should shift from more traditional satiety peptides towards motilin and/or ghrelin. It must be noted that all studies reported on *plasma* levels of GI peptides, pointing to systemic effects. Up to now, it is still unclear what the effect of taste receptor activation is on local secretion of GI peptides. It is conceivable that GI peptides are elevated at a splanchnic level or that they exert a more local or paracrine effect.

Only one study investigated brain signaling after post-oral delivery of non-caloric tastants. These authors found an increase in activity in homeostatic and hedonic brain regions and a decrease of activity in the brain stem and medulla after intragastric delivery of quinine [33]. These are interesting findings, since these data suggest additional mechanisms of effect of post-oral delivery of non-caloric tastants on eating behavior. It remains to be further elucidated whether these changes are mediated by orexigenic GI peptides.

### Implications of present data

In this review and meta-analysis, we have summarized the current knowledge on the effects of non-caloric tastants on energy intake. Using non-caloric tastants to reduce energy intake could provide a useful tool to combat the obesity epidemic.

Specifically, the use of bitter tastants appears to be promising. It is important to note that most studies described in this review have been performed in healthy adults with a normal BMI or in slightly overweight healthy adults. In several studies blunted postprandial levels of PYY [70, 71] and GLP-1 [72–74] were observed in obese subjects, and reductions in plasma CCK after weight loss [75, 76]. This points to alterations in the sensitivity of various receptors to GI peptides and in the magnitude of peptide secretion in obesity. Non-caloric tastants, because of their modest reduction in caloric intake, appear to be more suitable as a weight control intervention in a population that is worried about but has not yet gained excess weight.

This review and meta-analysis clearly illustrates the potential of bitter tastants in reducing caloric intake. However, several weaknesses of the currently available data should be elucidated. First, data are lacking uniformity. Most studies were underpowered: only small numbers of subjects have been included. Second, different study designs have been employed, making it difficult to compare results between various publications. An important variable with respect to energy intake reduction is the varying time interval between post-oral delivery of non-caloric tastants and meal intake. These intervals varied among studies between 30 min and 5 h but usually an interval around 60 min was chosen. The optimal interval between intervention and ad libitum meal is currently unknown, but standardization is necessary.

Third, most of the studies included in this review described the acute effects of post-oral delivery of non-caloric tastants on eating behavior. Only two studies described energy intake during the day [37, 39] and only one of those described an intervention period of 2 weeks [39]. Therefore, data on the long-term effects of post-oral delivery of non-caloric tastants are lacking. Consequently, it is unknown whether adaptation to the effects occurs. A fourth limitation is the lack of knowledge on the effects of post-oral delivery of umami tastants on eating behavior, as this was described in only one paper [42]. Fifth, only one study investigated several target locations in the GI tract [34]. Sixth, based on the present data it is unknown which bitter stimulus elicits the greatest effect on energy intake. Most studies described the use of either QHCl or DB.

### Future perspectives

To evaluate the effects of gastrointestinal delivery of non-caloric tastants on eating behavior, future studies should preferably standardize study design and doses of tastants. First, standardization could be achieved by creating consensus from the lead experts in the field. Second, mechanisms of effect should be more thoroughly investigated. For this, more research on the effects of post-oral delivery of non-caloric tastants on GI motility and systemic and local GI peptides secretion is needed. Based on the current data, we propose to focus on motilin and gastric motility. Third, compared with other tastants, knowledge on the effects of umami tastants is lacking. Therefore, the effects of post-oral delivery of umami tastants should be further elucidated. Fourth, taste receptors are expressed throughout the entire GI-tract [19–22]. The most appropriate location(s) for tastant delivery to modulate eating behavior is unknown. More research investigating delivery of tastants in different locations in the GI-tract is needed. Fifth, the optimal dose of tastants to exert an effect on eating behavior is unknown. Studies should focus on finding the balance between the maximum possible effect without occurrence of side effects. Sixth, it should be investigated which bitter stimulus elicits the largest effect on energy intake. For this, studies should compare different bitter stimuli. We propose to focus on QHCl and DB, as most data are available on these bitter stimuli and both stimuli activate a wide range of bitter receptors. Lastly, when all the foundations are laid out, the field should move towards implementation of the successful interventions in battling the obesity epidemic. For this, the longer-term effects of post-oral delivery of non-caloric tastants on energy intake and ultimately body weight control should be investigated.

## Conclusion

The current data show that, among tastants, bitter compounds are most effective in influencing eating behavior. Energy intake, in the acute setting, decreased modestly after post-oral delivery of bitter tastants. This highlights the potential preventive role of bitter tastants in battling the obesity epidemic. However, there are still several gaps in knowledge, for which recommendations have been provided. Systematically addressing these issues is warranted and worldwide collaboration could provide a welcome solution.

## Supplementary Information

Below is the link to the electronic supplementary material.

Supplementary file1 (PDF 43 KB)

Supplementary fig 1 Forest plot for pooled mean difference in energy intake after bitter components versus placebo. Pooled mean differences were calculated for the various combinations of doses of studies Bitarafan et al. 2019 [30] and Bitarafan et al. 2020 [29] respectively in order to eliminate a certain set of subjects having a large influence on the result: 37.5 mg QHCL and 275 mg QHCl (A), 37.5 mg QHCL and 600 mg QHCl (B), 75 mg QHCL and 275 mg QHCl (C), 75 mg QHCL and 600 mg QHCl (D), 225 mg QHCL and 275 mg QHCl (E), 225 mg QHCL and 600 mg QHCl (F). QHCl: quinine hydrochloride, DB: denatonium benzoate

Supplementary file1 (DOC 43 KB)
