# Supplementary material for: Effects of gastrointestinal delivery of non-caloric tastants on energy intake: a systematic review and meta-analysis
Source: Eur J Nutr. 2021 Feb 8;60(6):2923–47. doi: 10.1007/s00394-021-02485-4 (PMC8354866; doi:10.1007/s00394-021-02485-4)
Supplement: Supplementary file 1 — Supplementary file1 (PDF 43 KB) [file 394_2021_2485_MOESM1_ESM.pdf]

Effects of Gastrointestinal Delivery of Non-caloric Tastants on ~~Eating Behavior~~Energy Intake: A Systematic Review and Meta-Analysis

European Journal of Nutrition

Tim Klaassen[1,2], Daniel Keszthelyi[1], Freddy J. Troost[1,2], Aalt Bast[2], Adrian A.M. Masclee[1]

1. Division of Gastroenterology-Hepatology, Department of Internal Medicine, School of Nutrition and Translational Research in Metabolism (NUTRIM), Maastricht University Medical Center+, P.O. Box 5800, 6202 AZ Maastricht, The Netherlands
2. Food Innovation and Health, Center for Healthy Eating and Food Innovation, Maastricht University,5911 AA Venlo, The Netherlands

Corresponding author: T. Klaassen

E-mail: *t.klaassen@maastrichtuniversity.nl*

Postal address: Postbus 5800 | 6202 AZ Maastricht

Supplementary table 1 gives an overview of the results of all included articles.

Supplementary Table 1. Overview of included studies.

Taste	Reference	Subjects	Tastants and comparators used	Method of administration	Energy intake (Kcal)	GI symptoms and perceptions	Mechanisms of effect
**Sweet**	Rogers et al. (1990)[1] UK	12 subjects (6 men, 6 women, 18-26y, BMI 20.8)	Aspartame capsule (234 mg)	Gastric capsule	-175 Kcal	N/A	N/A

		Comparator:<br>Placebo capsule				
	15 subjects (10 men, 5 women, 19-24y, normal BMI	Aspartame capsule (235 mg)<br>Aspartame capsule (470 mg)<br>Comparator:<br>Placebo capsule	Gastric capsule	-138 Kcal for 235mg aspartame<br>-150 Kcal for 470 mg aspartame	Aspartame capsules reduced desire to eat and hunger scores ~~(p < .05)~~<br>Aspartame capsules tended to increase fullness compared with placebo (n.s.)	N/A
Black et al. (1993)[2]<br>Canada	18 subjects (18 men, 19-25y, BMI 21-25)	Aspartame capsule (340 mg)<br>Comparator:<br>Water	Gastric capsu le	No effect on energy intake<br>No effect on macronutrient composition	Aspartame capsules had no effect on appetite sensations	N/A
Little et al. (2009)[3]<br>UK	10 subjects	Saccharin (50 mg)<br>Aspartame (200 mg)<br>Comparator:<br>Tap water	Nasogastric catheter	N/A	No effects of aspartame or saccharin on hunger or fullness	No effects of aspartame or saccharin GE
Ma et al. (2009)[4]<br>Australia	7 subjects (24 y, BMI 21.6)	Sucralose (80 mg)<br>Sucralose (800 mg)<br>Comparator:	Nasogastric catheter	N/A	N/A	No effects of sucralose on gastric emptying, plasma glucose, plasma insulin, plasma GLP-1, or plasma GIP.

			Saline				
	Steinert et al. (2011)[5] Switzerland	12 subjects (6 men, 6 women, 23.3 y, BMI 23.0)	Aspartame (160 mg) Ace-K (200 mg) sucralose (62 mg) Comparator: Tap water	Nasogastric catheter	N/A	Artificial sweeteners reduced hunger, and increased satiety and fullness ratings to an intermediate amount between water and carbohydrate sugars (n.s.)	Sweeteners did not affect plasma GLP-1, PYY, ghrelin, glucose, insulin, or glucagon levels
	Van Avesaat et al. (2015)[6] The Netherlands	15 subjects (6 men, 9 women, 22.4 y, BMI 22.4)	Reb-A (540 mg) Comparator: Tap water	Nasoduodenal catheter	-24 Kcal (n.s.)	Reb-A did not influence appetite sensations. Reb-A did not induce GI symptoms	Reb-A did not affect plasma CCK, GLP-1, or PYY
	Wölnerhanssen et al. (2016)[7] Switzerland	20 subjects 10 lean subjects (5 men, 5 women, 26.6 y, BMI 21.7) 10 obese subjects (5 men, 5 women, 27.2 y, BMI 40.0)	Xylitol (50 g) Erythritol (75 g) Comparator: Tap water	Nasogastric catheter	N/A	Both sweeteners did not affect appetite sensations. Xylitol and erythritol led to bloating and diarrhea in 70% and 60% of subjects respectively (n.s.).	Gastric emptying was slowed during the first 60 mins after xylitol and erythritol vs. Control Plasma CCK, plasma GLP-1, Plasma glucose increased after xylitol and erythritol vs. control Plasma insulin increased after xylitol, but not after erythritol vs. control

	Meyer-Gerspach et al. (2018)[8] Belgium	12 subjects (6 men, 6 women, 23 y, BMI 23)	Ace-K (220 mg) Comparator: Tap water	Nasogastric catheter	N/A	Hunger: Strong initial decrease in hunger after Ace-K with a faster return of hunger after first time point and slower return of hunger in last part of curve after Ace-K vs. Control Satiation: Strong initial increase in satiation after Ace-K vs. control with faster decrease after first time point and slower decrease in last part of curve after Ace-K vs. control No adverse events	GI motility did not differ between Ace-K and control. A faster linear decrease in IGP from first post infusion time point, quicker return of IGP and quicker flattening of the curve during IGP recovery with faster return to baseline in last part of the IGP curve after Ace-K vs. control No effect of Ace-K on plasma motilin, octanoylated ghrelin, active GLP-1, CCK, gastrin, and glucose.
**Bitter**	Little et al. (2009)[3] UK	12 subjects	Naringin (~~1 mM~~290.27 mg) Quinine (~~0.198 mM~~32.2 mg) Comparator: Tap water	Nasogastric catheter	N/A	No effects of naringin or quinine on hunger or fullness scores compared with water	No effects of naringin or quinine on gastric emptying compared with water
	Andreozzi et al. (2015)[9] Italy	20 subjects (8 men, 12 women, 27 y, BMI 24)	QHCl capsule (18 mg)	Acid resistant capsules	-82 Kcal	QHCl did not affect satiety or desire to eat scores vs. Control. No adverse events	GE (evaluated in 8 subjects): no differences in GE between QHCl (87 mins) vs. Control (88 mins)

			Comparator: Placebo capsule				CCK: Higher ΔT90 vs T0 and ΔT90 vs T60 after QHCl vs. Control
	Avau et al. (2015)[10] Belgium	12 subjects (5 men, 30.6 y, BMI 23.8)	DB (~~1 μmol~~0.447 mg/kg body weight) Comparator: Saline	Nasogastric catheter	N/A	DB made subjects feel satiated earlier and at lower volumes during constant nutrient infusion No adverse effects	Less drop in IGP after DB
	Van Avesaat et al. (2015)[6] The Netherlands	15 subjects (6 men, 9 women, 22.4 y, BMI 22.4)	QHCl (75 mg) Comparator: Tap water	Nasoduodenal catheter	-44 Kcal (n.s.)	Quinine did not influence appetite sensations. Quinine did not induce GI symptoms	Quinine did not affect plasma CCK, GLP-1, or PYY levels.
	Mennella et al. (2016)[11] Italy	20 subjects (11 men, 9 women, 25.3 y, BMI 22.1	Microencapsulated bitter secoiridoids (100 mg) Comparator: Coating only	Microencapsulation to mask oral tasting. Exact location of effect in GI tract unknown	Lunch: - 88 Kcal (n.s.) Post-lunch: -252 Kcal 24h energy intake: - 340 Kcal	no effect of bitter encapsulate on fullness, satiety, hunger or desire to eat	Bitter encapsulate increased plasma GLP-1 30 mins after intervention, but had no effect on blood glucose, plasma amylin, plasma ghrelin, plasma glucagon, plasma GIP, plasma insulin, plasma leptin, plasma PP, or plasma PYY vs. Control

	Peters et al. (2016)[12] The Netherlands	57 subjects (all women, 40.5 y, BMI 26.5)	Bitter mixture containing: Raisin ~~flower~~ flavor (22.0 mg) Sucrose Octa Acetate (0.88 mg) Quassia extract (0.088 mg) Comparator: Placebo capsule	Intragastric capsule, 2 times daily for 14 days	Day 0 vs. day 14: Meals only: -109 Kcal (n.s.) Meals + snack: -86 Kcal (n.s.) Breakfast: -30 Kcal (n.s.) Lunch: -61 Kcal (n.s.) Dinner: -1 Kcal (n.s.) Snacks: +41 Kcal (n.s.)	N/A	N/A
	Deloose et al. (2017)[13] Belgium	20 subjects (10 men, 10 women, 27 y, BMI 24)	DB (~~1 µmol~~0.447 mg/Kg body weight) Comparator: Tap water	Nasogastric catheter	N/A	Women: Switch from gastric to duodenal phase 3 origin was accompanied by lower percentage change of hunger scores after DB vs. Control Men: Percentage change in hunger scores during phase 3 contraction did not differ after DB vs. Control (n.s.) No adverse events after DB administration	Women: DB reduced number of gastric phase 3 contractions from 67% (control) to 33% (DB) in women. Interval between IG administration and occurrence of phase 3 did not differ between control (76 min) and DB (93 min) in women (n.s.). Men: No difference in origin of phase 3 contractions between control (57%

							gastric) and DB (40% gastric) in men (n.s.). Interval between IG administration and occurrence of phase 3 did not differ between control (76 min) and DB (111 min) in men (n.s.).
		12 subjects (all women, 31 y, BMI 22)	DB (~~1 µmol~~0.447 mg/Kg body weight) Comparator: Tap water	Nasogastric catheter	N/A	No adverse events after DB administration	Plasma motilin was lower after DB vs. Control. No differences between plasma total ghrelin or octanoylated ghrelin after DB vs. Control
		13 subjects (all women, 28 y, BMI 23)	DB (~~1 µmol~~0.447 mg/Kg body weight) Comparator: Tap water	Nasogastric catheter	N/A	Hunger scores after a standardized meal were lower after DB vs. Control. Satiety scores were higher after a standardized meal after DB. No adverse events after DB administration	GE (measured in 6 subjects) did not differ between control and DB (both 109 mins.
		20 subjects (all women, 23 y, BMI 22)	DB (~~1 µmol~~0.447 mg/Kg body weight) Comparator:	Nasogastric catheter	-76 Kcal (n.s.)	No adverse events after DB administration	N/A

			Tap water				
	Deloose et al. (2018)[14] Belgium	10 subjects (10 women, 33 y, BMI 22)	QHCl (~~10 µmol~~3.6 mg/kg body weight) Comparator: Milli-Q water	Nasogastric catheter	N/A	No adverse events	Plasma motilin and plasma ghrelin levels decreased after QHCl. No difference in plasma octanoylated ghrelin levels Time* treatment effect for antral motility. No main effect of treatment. No effects of QHCl on duodenal motility.
	Bitarafan et al. (2019)[15] Australia	14 subjects (14 men, 25 y, BMI 22.5)	QHCl (37.5 mg, Q37.5)) QHCl (75 mg, Q75)) QHCl (225 mg, Q225)) Comparator: Saline	Nasoduodenal catheter	Q37.5: - 31Kcal (n.s.), Q75: - 59 Kcal (n.s.), Q225: -11 Kcal (n.s.)	No differences in VAS scores for hunger, desire to eat, prospective consumption, or fullness after Q37.5, Q75, or Q225 vs. Control. No adverse events, no effects of Q37.5, Q75, or Q225 on nausea or bloating.	No effect of Q37.5, Q75, or Q225 on antral pressure waves, basal pyloric pressure, isolated pyloric pressure waves, and duodenal pressure waves vs. Control. No effects of Q37.5, Q75, and Q225 on plasma CCK or blood glucose vs. Control.
	Iven et al. (2019)[16] Belgium	16 subjects (16 women, 24.5 y, BMI 21.9)	QHCl (~~10 µmo~~3.6 mg/Kg body weigh)	Nasogastric catheter	-67.6 Kcal	Hunger scores increased after control and decreased after QHCl (n.s.)	Decreases in total ghrelin, octanoylated ghrelin, and motilin after QHCl vs. control

			Comparator:<br>Milli-Q water			Prospective food consumption scores decreased after QHCl vs. Control<br>Satiety scores increased after QHCl vs. Control<br>fullness scores increased after QHCl vs. control<br>Minimal nausea scores reported	Brain activity in homeostatic and hedonic regions:<br>Increased activity after QHCl vs. Control in anterior insula, ACC, amygdala, putamen, nucleus accumbens, pallidum, caudate head and caudate body, medial and lateral OFC, hypothalamus and midbrain.<br>Decreased activity in brainstem/medulla
	Walker et al. (2019)[17]<br>New Zealand	30 subjects (30 men, 24y, BMI 23.1)	Amarasate extract capsule (500 mg, HD)<br>Amarasate extract capsule (200 mg, LD)<br>Comparator:<br>Placebo capsule	Acid resistant capsule	N/A	From T=90 onwards HD and LD show lower mean changes in hunger and fullness<br>Lower mean changes in fullness for HD from t=120 onwards, only t=180 and t=330 for LD.<br>No nausea. 3 participants in HD and 1 in LD had liquid loose bowel movements	N/A

	Bitarafan et al. (2020)[18] Australia	15 subjects (15 men, 26 y, BMI 23.2)	QHCl (275 mg, Q275)<br>QHCl (600 mg, Q600)<br>Comparator: Saline	Nasogastric catheter	N/A	No effects of Q275 or Q600 on hunger, desire to eat, prospective consumption, or fullness scores.<br>No effects of Q275 or Q600 on bloating or nausea vs. Control. No other adverse effects	No effects of Q275 or Q600 on gastric emptying.<br>Plasma insulin was increased 30 mins after Q275 and Q600 vs. Control.<br>No effects of Q275 or Q600 on plasma glucose, plasma glucagon, or plasma GLP-1.<br>After mixed nutrient drink:<br>Q275 and Q600 lowered glucose<br>Q275 and Q600 increased plasma insulin<br>No difference in glucagon response after nutrient drink.<br>Q275 increased plasma GLP-1, Q600 did not.
		12 subjects (12 men, 26 y, BMI 23.1)	QHCl (275 mg, Q275)<br>QHCl (600 mg, Q600)<br>Comparator:	Nasogastric catheter	No effect of treatment on energy intake Q275: +26 Kcal, Q600: -53 Kcal	No effects of Q275 or Q600 on hunger, desire to eat, prospective consumption, or fullness scores.	N/A

			Saline			No effects of Q275 or Q600 on bloating or nausea vs. Control. No other adverse effects	
**Umami**	Van Avesaat et al. (2015)[6] The Netherlands	15 subjects (6 men, 9 women, 22.4 y, BMI 22.4)	MSG (2 g) Comparator: Tap water	Intraduodenal catheter	+ 5 Kcal (n.s.)	MSG decreased hunger and desire to eat, but did not influence satiation or fullness. MSG did not induce GI symptoms	Monosodium glutamate did not affect plasma CCK, GLP-1, or PYY levels.
**Combination**	Van Avesaat et al. (2015)[6] The Netherlands	15 subjects (6 men, 9 women, 22.4 y, BMI 22.4)	Tastant mixture: Reb-A (540 mg) QHCl (75 mg) MSG (2 g) Comparator: Tap water	Nasoduodenal catheter	-64 Kcal	The tastant mixture decreased hunger and desire to eat, but not satiation or fullness. The tastant mixture did not induce GI symptoms	The tastant mixture did not affect plasma CCK, GLP-1, or PYY levels.
	Klaassen et al. (2019)[19] The Netherlands	14 subjects (3 men, 11 women, 25.6 y, BMI 22.3)	Tastant mixture: Reb-A (540 mg) QHCl (75 mg) MSG (2 g) Comparator: Tap water	Naso-duodenal-ileal catheter	Duodenal +16.7 Kcal (n.s.), ileal +28.1 Kcal (n.s.), combined duodenal and ileal +31.5 Kcal (n.s.)	No effects of duodenal-, ileal- or combined duodenal and ileal taste receptor activation on appetite sensations. The tastant mixture did not induce GI symptoms	N/A

y: years, BMI: body mass index, N/A: not applicable, n.s.: not significant, GE: gastric emptying, GLP-1: glucagon like peptide 1, GIP: glucose-dependent insulinotropic polypeptide, Ace-K: acesulfame potassium, PYY: peptide yy, Reb-A: rebaudioside A, GI: gastrointestinal, CCK: cholecystokinin, IGP: intragastric pressure, QHCL: quinine hydrochloride, DB: denatonium benzoate, PP: pancreatic polypeptide, IG: intragastric, ACC: anterior cingulate cortex, OFC: orbitofrontal cortex, MSG: monosodium glutamate

1. Rogers PJ, Pleming HC, Blundell JE. Aspartame Ingested without Tasting Inhibits Hunger and Food-Intake. Physiology & Behavior. 1990;47(6):1239-43.
2. Black RM, Leiter LA, Anderson GH. Consuming aspartame with and without taste: differential effects on appetite and food intake of young adult males. Physiol Behav. 1993;53(3):459-66.
3. Little TJ, Gupta N, Case RM, Thompson DG, McLaughlin JT. Sweetness and bitterness taste of meals per se does not mediate gastric emptying in humans. Am J Physiol Regul Integr Comp Physiol. 2009;297(3):R632-9.
4. Ma J, Bellon M, Wishart JM, et al. Effect of the artificial sweetener, sucralose, on gastric emptying and incretin hormone release in healthy subjects. Am J Physiol Gastrointest Liver Physiol. 2009;296(4):G735-9.
5. Steinert RE, Frey F, Topfer A, Drewe J, Beglinger C. Effects of carbohydrate sugars and artificial sweeteners on appetite and the secretion of gastrointestinal satiety peptides. Br J Nutr. 2011;105(9):1320-8.
6. van Avesaat M, Troost FJ, Ripken D, Peters J, Hendriks HF, Masclee AA. Intraduodenal infusion of a combination of tastants decreases food intake in humans. Am J Clin Nutr. 2015;102(4):729-35.
7. Wolnerhanssen BK, Cajacob L, Keller N, et al. Gut hormone secretion, gastric emptying, and glycemic responses to erythritol and xylitol in lean and obese subjects. Am J Physiol Endocrinol Metab. 2016;310(11):E1053-61.
8. Meyer-Gerspach AC, Biesiekierski JR, Deloose E, et al. Effects of caloric and noncaloric sweeteners on antroduodenal motility, gastrointestinal hormone secretion and appetite-related sensations in healthy subjects. Am J Clin Nutr. 2018;107(5):707-16.
9. Andreozzi P, Sarnelli G, Pesce M, et al. The Bitter Taste Receptor Agonist Quinine Reduces Calorie Intake and Increases the Postprandial Release of Cholecystokinin in Healthy Subjects. Journal of Neurogastroenterology and Motility. 2015;21(4):511-9.
10. Avau B, Rotondo A, Thijs T, et al. Targeting extra-oral bitter taste receptors modulates gastrointestinal motility with effects on satiation. Sci Rep. 2015;5:15985.
11. Mennella I, Fogliano V, Ferracane R, Arlorio M, Pattarino F, Vitaglione P. Microencapsulated bitter compounds (from Gentiana lutea) reduce daily energy intakes in humans. Br J Nutr. 2016:1-10.
12. Peters HPF, Koppenol W, Schuring EAH, Gouka R, Mela DJ, Blom WAM. The effect of two weeks ingestion of a bitter tastant mixture on energy intake in overweight females. Appetite. 2016;107:268-73.

13. Deloose E, Janssen P, Corsetti M, et al. Intragastric infusion of denatonium benzoate attenuates interdigestive gastric motility and hunger scores in healthy female volunteers. Am J Clin Nutr. 2017;105(3):580-8.
14. Deloose E, Corsetti M, Van Oudenhove L, Depoortere I, Tack J. Intragastric infusion of the bitter tastant quinine suppresses hormone release and antral motility during the fasting state in healthy female volunteers. Neurogastroenterology and Motility. 2018;30(1).
15. Bitarafan V, Fitzgerald PCE, Little TJ, et al. Effects of Intraduodenal Infusion of the Bitter Tastant, Quinine, on Antropyloroduodenal Motility, Plasma Cholecystokinin, and Energy Intake in Healthy Men. Journal of Neurogastroenterology and Motility. 2019;25(3):413-22.
16. Iven J, Biesiekierski JR, Zhao DX, et al. Intragastric quinine administration decreases hedonic eating in healthy women through peptide-mediated gut-brain signaling mechanisms. Nutritional Neuroscience. 2019;22(12):850-62.
17. Walker E, Lo K, Tham S, et al. New Zealand Bitter Hops Extract Reduces Hunger During a 24 h Water Only Fast. Nutrients. 2019;11(11).
18. Bitarafan V, Fitzgerald PCE, Little TJ, et al. Intragastric administration of the bitter tastant quinine lowers the glycemic response to a nutrient drink without slowing gastric emptying in healthy men. Am J Physiol Regul Integr Comp Physiol. 2020;318(2):R263-R73.
19. Klaassen T, Alleleyn AME, van Avesaat M, Troost FJ, Keszthelyi D, Masclee AAM. Intraintestinal Delivery of Tastants Using a Naso-Duodenal-Ileal Catheter Does Not Influence Food Intake or Satiety. Nutrients. 2019;11(2).
